# Exploring the Molluscan Microbiome: Diversity, Function, and Ecological Implications

**DOI:** 10.3390/biology14081086

**Published:** 2025-08-20

**Authors:** Tsireledzo Goodwill Makwarela, Nimmi Seoraj-Pillai, Tshifhiwa Constance Nangammbi

**Affiliations:** Department of Nature Conservation, Faculty of Science, Tshwane University of Technology, Staatsartillerie Rd, Pretoria West, Pretoria 0183, South Africa; seorajpillayn@tut.ac.za (N.S.-P.); nangammbitc@tut.ac.za (T.C.N.)

**Keywords:** microbiome, host–microbe interaction, functional redundancy, gut bacteria, symbiosis, aquaculture, microbial diversity, antimicrobial resistance, core microbiota, environmental stress

## Abstract

Mollusks are among the most ecologically and economically significant invertebrates; yet, their associated microbiomes remain understudied relative to those of other metazoans. This scoping review synthesizes the current literature on the diversity, composition, functional roles, and ecological implications of molluscan microbiomes, with an emphasis on three major groups: gastropods, bivalves, and cephalopods. Drawing on studies from terrestrial, freshwater, and marine systems, we identified the dominant bacterial phyla, including Proteobacteria, Bacteroidetes, and Firmicutes, and explored how microbiota vary across different habitats, diets, tissue types, and host taxonomies. We examined the contribution of molluscan microbiomes to host functions, including digestion, immune modulation, stress responses, and nutrient cycling. Particular attention was given to the role of microbiota in shell formation, pollutant degradation, and adaptation to environmental stressors. The review also evaluated microbial interactions at different developmental stages and under aquaculture conditions. Factors influencing microbiome assembly, such as the host’s genetics, life history traits, and environmental exposure, were mapped using conceptual and graphical tools. Applications of molluscan microbiome research in aquaculture, conservation biology, and environmental biomonitoring are highlighted. However, inconsistencies in the sampling methods, taxonomic focus, and functional annotations limit the generalizability across taxa. We identify key knowledge gaps and propose future directions, including the use of meta-omics, standardized protocols, and experimental validation to deepen insights. By synthesizing emerging findings, this review contributes to a growing framework for understanding mollusk–microbiome interactions and their relevance to host fitness and ecosystem health. It further establishes the importance of mollusks as model systems for advancing microbiome science.

## Simple Summary

Mollusks, including snails, mussels, and clams, are ecologically and economically important animals that interact with a diverse array of microorganisms living on and within their bodies. These microbial communities play key roles in digestion, nutrient processing, immune support, and the ability of mollusks to adapt to environmental stress. This review examines the current understanding of these host–microbe relationships, highlighting how microbial diversity varies across species, body parts, and environmental conditions. It also examines how factors such as the temperature, food availability, and pollution can impact the microbial balance, potentially leading to health issues. The findings show that mollusks and their microbial partners form highly specialized systems that can serve as indicators of ecosystem health. Studying these interactions not only deepens our knowledge of animal biology and ecology but also provides valuable insights for sustainable aquaculture, environmental monitoring, and conservation planning.

## 1. Introduction

Mollusks, including major groups such as bivalves (e.g., oysters and clams) and gastropods (e.g., snails and slugs), harbor specialized gut microbial communities that facilitate nutrient absorption, energy metabolism, and protection against pathogens. For example, the core bacterial taxa in gastropods have been shown to persist across generations, suggesting co-evolutionary patterns and functional stability in host–microbe associations [1]. These microbial communities play a crucial role in the ecological success of mollusks, enabling them to thrive in diverse and often fluctuating environments. The composition and functional potential of molluscan microbiomes are influenced by the host’s phylogeny, the habitat type, and dietary behavior [2]. The phenomenon of phylosymbiosis, whereby closely related host species share similar microbial assemblages, highlights the evolutionary imprint on microbiome structure and function. Such complexity underscores the need for advanced molecular approaches, including metagenomics and metabarcoding, to profile microbial diversity and assess host-specific microbial functions accurately [2]. Environmental change adds a layer of complexity to host–microbiome dynamics. Anthropogenic stressors, such as ocean warming, pollution, and microplastic accumulation, have been implicated in altering molluscan microbiomes, often leading to dysbiosis—a condition characterized by an imbalance in microbial communities that is associated with increased vulnerability to pathogens [3,4]. These disruptions not only threaten molluscan health but may also compromise ecosystem services provided by mollusks. Beyond individual host effects, molluscan microbiomes play a crucial role in broader ecological processes, including nutrient cycling and water purification. For instance, bivalve-associated microbes play key roles in nitrogen transformation within aquatic sediments, facilitating vertical nutrient flux and ecosystem-level biogeochemical cycling [5]. These microbially mediated processes position mollusks and their symbionts as valuable bioindicators of ecosystem integrity and environmental change. The emergence of pathogenic bacteria within molluscan microbiomes has raised concerns about disease outbreaks in aquaculture and public health risks linked to seafood consumption. Studies, such as those by Cutarelli et al. [6], have linked microbial dysbiosis to increased disease prevalence in economically important species, including the Mediterranean mussel. These findings highlight the importance of continued microbial monitoring, particularly in environments experiencing nutrient enrichment and harmful algal blooms [3]. Technological advancements in multi-omics, such as metaproteomics and metabolomics, offer unprecedented opportunities to unravel the functional capacities of molluscan microbiota. These approaches can reveal the microbial contributions to host metabolism, stress tolerance, and immunity, offering promising applications in aquaculture, conservation, and environmental monitoring [7].

The ecological niche and lifestyle of mollusks are fundamental determinants of their associated microbiomes, with significant implications for ecological functions and food safety. Mollusks occupy diverse environments—planktonic, benthic, estuarine, freshwater, and terrestrial—that shape their microbial assemblages through differing exposure routes and host–microbe dynamics. Planktonic species, which drift freely in open water, are subject to microbial colonization from ambient microbial pools, while benthic mollusks such as *Mytilus* and *Crassostrea* acquire microbiota from sediment-associated communities, often exhibiting environment-driven microbiome structures [2,8]. Terrestrial and aquatic snails harbor distinct microbiomes shaped by their environments and behaviors. Terrestrial snails like Achatina have microbiomes influenced by the soil, plants, and surface microbes [9,10], while freshwater environments and associated organisms shape aquatic snails like Biomphalaria [11]. Evidence suggests that vertical transmission of microbes is more pronounced in some terrestrial snail groups compared to their aquatic counterparts [12]. These microbiomes contribute to host nutrition, immune modulation, and resilience to pathogens, with lifestyle-mediated differences in the microbial composition affecting the safety of mollusks consumed by humans [13,14]. For instance, mollusks exposed to anthropogenic stressors, including pollution and aquaculture runoff, show shifts in microbial diversity and an increase in opportunistic or pathogenic taxa, underscoring the need to understand microbiome–lifestyle interactions to improve food safety and ecological monitoring [15,16]. This review adopts a narrative approach, drawing on a critical synthesis of peer-reviewed studies across diverse molluscan taxa and habitats. Rather than following a systematic review framework, it integrates the recent literature to explore patterns of microbial diversity, functional roles, and ecological interactions within molluscan microbiomes, with particular attention to food safety implications and environmental sensitivity.

## 2. Diversity and Composition of Molluscan Microbiomes

### 2.1. Gastropods

Gastropods, the most speciose and ecologically versatile class within Mollusca, host complex microbiomes that vary significantly with their habitat, diet, and environmental conditions. Recent studies using high-throughput sequencing have uncovered diverse microbial communities within the gastrointestinal tracts of both aquatic and terrestrial species, reflecting intricate host–microbe relationships shaped by their ecological niche [17,18]. For instance, *Achatina fulica* (Bowdich, 1822) and *Biomphalaria pfeifferi* (Krauss, 1848) consistently harbor bacterial taxa dominated by Proteobacteria, which are implicated in nutrient assimilation, metabolic processing, and immune regulation. Beyond the phylum-level patterns, conserved genera such as *Cloacibacterium* and *Aeromonas* Stanier, 1943, have been identified across multiple gastropod lineages [1], with evidence suggesting vertical transmission and long-term functional integration. These core taxa have been linked to critical roles in lignocellulose degradation, immune modulation, and pathogen exclusion [18,19], particularly in detritivorous species such as *Arion ater* (Linnaeus, 1758). Environmental factors strongly influence the microbiome composition. In hydrothermal vent gastropods, the microbial assemblages are highly specialized and shaped by local physicochemical gradients, supporting host survival in extreme conditions [20,21]. Conversely, anthropogenic pressures such as pollution and aquaculture runoff are associated with reductions in microbial diversity and the proliferation of opportunistic taxa, potentially disrupting mutualistic interactions [22,23]. These patterns suggest that gastropod-associated microbiomes are sensitive ecological indicators. However, the current studies disproportionately focus on a few taxa and geographic regions. For example, ref. [12] demonstrated distinct microbial assemblages between terrestrial and aquatic snail species, underscoring the importance of broadening the taxonomic and habitat representation in future microbiome surveys. The incorporation of functional data, including from metagenomic and transcriptomic analyses, is also necessary to move beyond taxonomic inventories and critically evaluate the contributions of microbes to host fitness and ecosystem functioning.

### 2.2. Bivalves

Bivalves, including mussels, oysters, and clams, are foundational to aquatic ecosystems and the global aquaculture industry. These filter-feeding mollusks host diverse microbiomes that contribute to key physiological and ecological processes, including digestion, immune function, and environmental detoxification. Microbial communities inhabit multiple tissue compartments, such as the gut, gills, and hemolymph, with their composition varying by the tissue type, host species, and environmental context [24,25]. Environmental factors such as the temperature, salinity, and geographic location significantly influence microbiome diversity. For instance, *Unio crassus* Philipsson, 1788, a freshwater mussel, exhibits site-specific microbial profiles, indicating that the local environmental conditions influence the shaping of gut communities [24]. Similarly, *Mytilus galloprovincialis* Lamarck, 1819, a common intertidal marine mussel, exhibits habitat-dependent variation in its microbiome composition [25].

Across bivalve species, members of the Proteobacteria and Firmicutes phyla dominate microbial communities and are associated with symbiotic roles in nutrient metabolism and host defense [26,27]. Mollicutes, notably observed in *Crassostrea virginica* Gmelin, 1791, and *Mytilus edulis* Linnaeus, 1758, are implicated in maintaining host tissue homeostasis, although their functional roles remain underexplored [27,28]. Notably, tissue-specific variation is consistent across hosts, underscoring the anatomical compartmentalization of microbial function. Microbiota contribute to immune priming, nitrogen cycling, and resistance to pathogens; yet, these communities are sensitive to environmental disruptions. Climate-related stressors, such as ocean acidification and warming, have been linked to microbial dysbiosis, resulting in reduced diversity and compromised host health [3,4,29]. Pollution and pathogen exposure can shift microbial profiles toward opportunistic or pathogenic taxa, diminishing beneficial symbioses [30,31]. These findings position the bivalve microbiome as a bioindicator for environmental disturbance and a potential target for ensuring sustainable aquaculture practices. However, further integration of metagenomic and functional data is needed to move beyond taxonomic inventories and fully understand host–microbe–environment dynamics.

### 2.3. Cephalopods

Cephalopods, such as squids, octopuses, and cuttlefish, represent one of the most evolutionarily advanced molluscan classes, distinguished by their complex behavior, neural sophistication, and dynamic physiology. Despite their ecological and biomedical importance, research on the microbiome of cephalopods remains limited compared to that on gastropods and bivalves. However, emerging studies indicate that cephalopods host distinctive microbial communities shaped by a combination of their phylogenetic history, diet, and habitat [2]. The gut microbiota of *Octopus vulgaris* Cuvier, 1797, and related species is dominated by Alphaproteobacteria and Gammaproteobacteria, bacterial groups associated with nutrient absorption, metabolic regulation, and immune modulation [32]. Comparative analyses suggest that evolutionary divergence among cephalopods contributes to distinct microbial profiles, although data from cross-species comparisons remain sparse. Beyond the digestive system, microbial populations are also found in the skin, mantle, and reproductive tissues, exhibiting tissue-specific variability and sex-based differences [33]. These populations have been identified as exhibiting sexually dimorphic patterns in microbial diversity, indicating a potential link between the microbiome composition and reproductive physiology. These patterns suggest broader physiological roles for microbial symbionts, including potential influences on reproductive biology, mating behavior, and host adaptability [2].

Environmental factors such as ocean warming, pollution, and habitat degradation disrupt cephalopod microbiomes, often leading to dysbiosis and functional impairments [34,35]. The loss of beneficial symbionts or the expansion of opportunistic taxa under stress conditions may reduce hosts’ resilience and compromise their ecological fitness. Functional insights, though limited, suggest that microbiomes actively support stress tolerance, immune priming, and metabolic homeostasis, underscoring their importance in survival strategies during environmental change [36]. The current understanding of cephalopod–microbiome interactions remains fragmented due to the limited number of species studied and the lack of integrative multi-omics approaches. Future research should prioritize comparative studies across species and environments, as well as functional validation of microbial roles using experimental methods. Molluscan microbiomes comprise diverse bacterial communities that vary by the host species and tissue type. To summarize these differences, Table 1 presents the dominant bacterial taxa associated with key mollusk hosts and their putative functional roles, environmental sensitivities, and representative references. This overview highlights the ecological significance of these microbial assemblages and their potential contributions to the host’s physiology across major molluscan groups.

### 2.4. Tissue-Specific Microbiomes

Recent studies have increasingly highlighted the significance of tissue-specific microbiomes in cephalopods, revealing how microbial communities vary across anatomical sites and contribute to the host’s physiology. Distinct microbial assemblages have been identified in the skin, gut, and reproductive organs, with each compartment supporting functionally specialized symbionts. These tissue-associated microbiomes appear to influence key processes, including immunity, nutrient absorption, and reproduction. The skin microbiome in *O. vulgaris* demonstrates considerable variation, with evidence suggesting sex-dependent differences in the microbial composition [33]. The presence of antimicrobial compounds in cephalopod ink is also thought to modulate skin-associated microbial populations, further supporting the existence of a functional role for these communities in pathogen defense [32]. However, the precise mechanisms regulating skin–microbiota interactions remain underexplored, warranting further investigation into their functional significance.

**Figure 1:**
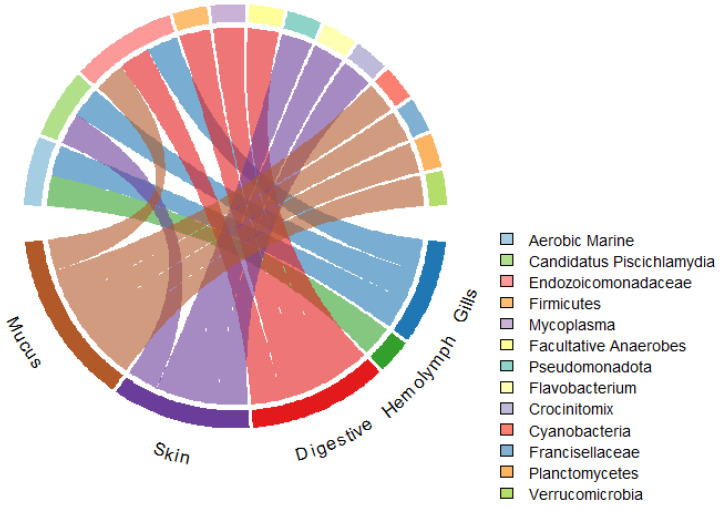
Tissue–microbe associations in mollusks.

In the gut, microbiota support digestive efficiency and metabolic adaptation. Species-specific diets and environmental exposure shape these communities [2]. For example, in *Sepia officinalis* Linnaeus, 1758, gut microbes have been linked to enhanced survival under starvation conditions, reflecting a role in nutritional plasticity [42,43]. The dominance of bacteria capable of degrading complex substrates further underscores their contribution to energy acquisition in dynamic marine environments. Reproductive tissues also harbor specialized microbiota, though this area remains relatively understudied. Emerging evidence suggests that these microbes may influence reproductive success, embryo development, or vertical transmission of beneficial taxa, hinting at co-evolved host–microbe partnerships [2,32]. Importantly, tissue-specific microbial communities are sensitive to environmental stressors. Exposure to pollutants, temperature anomalies, and heavy metals has been shown to disrupt the microbiome structure across the skin, gut, and reproductive systems [44,45]. Such disruptions may impair immune defense, reduce the host’s fitness, and compromise reproductive outcomes. As cephalopods increasingly serve as bioindicators of marine health, understanding the integrity of their tissue-specific microbiomes offers valuable insights into the impacts of anthropogenic change on marine biodiversity. The microbial assemblages in mollusks demonstrate clear tissue-specific patterns, with certain genera shared across multiple tissues while others show strict compartmentalization. Figure 1 illustrates these associations through a chord diagram mapping the dominant bacterial genera to specific tissue types across various molluscan hosts. Notably, *Mycoplasma* appears across all tissues, whereas taxa like *Lactobacillus* and *Gardnerella* are more restricted, reflecting specialized symbiotic relationships as discussed in recent studies [3,46,47,48,49].

### 2.5. Core Microbiota and Host Specificity

Recent research has identified consistent core microbial taxa in mollusks, particularly in cephalopods such as *O. vulgaris*. High-throughput sequencing approaches have revealed genera like *Photobacterium* Beijerinck, 1889, and *Mycoplasma* Nowak, 1929, to be persistent members of the gastrointestinal microbiome across individuals, highlighting their potential foundational roles in the host’s physiology [2,50]. These taxa are involved in critical functions, such as nutrient metabolism, immune modulation, and pathogen suppression, all of which are essential for maintaining host resilience in dynamic marine environments. Bivalves also possess well-defined core microbiota. In *M. edulis* (blue mussel), the dominant bacterial communities, which are frequently composed of members from Firmicutes and Proteobacteria, facilitate digestion and offer protection against opportunistic pathogens [51,52]. These core microbiota exhibit temporal and spatial stability, suggesting their ecological importance and co-evolution with host systems [53,54].

However, while relatively stable, the core microbiomes are not immune to environmental perturbations. Studies have shown that pollutant exposure can disrupt the core community structure in bivalves, reducing diversity and altering functional profiles, often with negative consequences for host health [6]. Similarly, salinity fluctuations have been linked to shifts in the microbial composition, indicating that environmental plasticity remains a vital trait even among conserved microbial assemblages [55]. A key feature of the core microbiota in mollusks is their host specificity. Comparative studies across oysters, clams, mussels, and cephalopods demonstrate that distinct microbial signatures are shaped by the host’s phylogeny, diet, habitat, and evolutionary history [2,31]. This specificity suggests that host–microbe co-evolution has led to tailored microbial consortia that enhance fitness, influence behavior, and potentially contribute to niche differentiation. Understanding these associations is therefore essential not only for studying microbiome ecology but also for informing conservation strategies and aquaculture practices. Several key features influence the assembly and stability of molluscan microbiomes. These include the host species identity, phylogeny, habitat, diet, and environmental stressors, each exerting selective pressures that shape the microbial diversity and function. Table 2 summarizes these features along with illustrative examples from the literature. Understanding these factors is critical for deciphering microbiome–host interactions and their ecological implications.

Quantitative diversity indices such as Shannon’s diversity index (H′) and Simpson’s index (D) have emerged as essential tools for evaluating the richness and evenness of microbial communities in mollusks. These metrics provide valuable insights into the ecological structure and functional stability of host-associated microbiota. For example, studies on the gut microbiota of *M. galloprovincialis* have reported H′ values ranging from approximately 2.8 to 4.1, indicating moderate microbial diversity influenced by environmental parameters such as the water temperature, salinity, and nutrient load [4,29,66]. These findings underscore the capacity of benthic marine organisms to maintain adaptable and resilient microbiomes in response to ecological fluctuations.

In contrast, cephalopod reproductive tissues typically harbor less diverse microbial communities, often dominated by *Mycoplasma*, resulting in lower Shannon and Simpson diversity values [67,68]. This reduced diversity likely reflects a high degree of functional specialization and selective colonization, in stark contrast to the more compositionally complex gut microbiomes. Comparative analyses across tissue types have consistently identified the gut as the most diverse microbial niche, followed by the skin and reproductive tract [54,69]. Such hierarchical patterns suggest that microbial diversity is tightly linked to tissue function and exposure to external environmental gradients.

Moreover, the use of Simpson’s index has proven particularly effective in detecting low evenness in specialized tissues such as the reproductive tract, where a few dominant taxa prevail [68,70]. These trends emphasize the influence of the host’s physiology and anatomical compartmentalization on the microbial community composition. Collectively, the application of diversity indices provides a quantitative foundation for understanding microbial ecosystem functions, host–microbe co-adaptation, and the ecological implications of microbiome variation within marine invertebrates.

## 3. Environmental and Host-Related Drivers of Microbiome Structure

### 3.1. Temperature, Oxygen, and pH

Abiotic factors such as the temperature, oxygen availability, and pH are significant determinants of the molluscan microbiome’s structure and function. The temperature is widely recognized as one of the most potent drivers of microbiome variability. It influences microbial growth rates, gene expression, and enzymatic processes, which collectively reshape community structures. Shifts in the temperature can induce significant changes in a microbial community’s structure and diversity. Rocca et al. [71] reported that microbial diversity often follows unimodal patterns along thermal gradients, indicating that microbial communities exhibit optimal diversity within specific temperature ranges and decline in diversity outside these thresholds. In marine bivalves, elevated temperatures have been shown to reduce the microbial richness, particularly in the hemolymph, leading to compromised immune function and metabolic imbalances [29]. Moreover, thermal stress is increasingly linked to microbial dysbiosis, which may impair resilience and predispose hosts to opportunistic infections, particularly under climate change scenarios [72]. The oxygen availability interacts closely with the temperature to influence aquatic microbiomes. In stratified or hypoxic environments, distinct microbial consortia emerge, often characterized by anaerobic or facultatively anaerobic taxa. For mollusks, especially those inhabiting sediment-rich or eutrophic habitats, fluctuations in the oxygen levels can alter the microbiome functionality, particularly in processes such as detoxification, nitrogen cycling, and pathogen resistance [26,73]. These shifts may affect the host’s performance and survival, particularly during periods of thermal or hypoxic stress. The pH is another critical factor that shapes molluscan microbiomes by modulating nutrient solubility, enzymatic function, and microbial metabolism. Aquatic acidification, driven by CO_2_ emissions or pollution, can alter the stability and composition of microbial communities. In mollusk-associated microbiomes, pH fluctuations influence both the bacterial community structure and the expression of genes involved in essential metabolic pathways [74]. For bivalves and gastropods, exposure to a low or unstable pH has been shown to disturb beneficial symbiotic relationships, potentially impairing shell formation, digestion, and immune responses [27]. Together, these abiotic factors act as key ecological filters, determining which microbial taxa persist within the host and how they function. Understanding these dynamics is crucial for predicting molluscan responses to environmental change and designing microbiome-informed conservation or aquaculture strategies.

### 3.2. Host’s Diet and Development

Diet and ontogenetic development are pivotal determinants of the microbiome structure and function across molluscan taxa. These host-related factors modulate not only the microbial diversity and community composition but also impact the host’s physiology, immunity, and resilience to environmental fluctuations. Dietary intake has a direct influence on the microbiomes of both filter-feeding and carnivorous mollusks. In bivalves, which rely heavily on suspended organic matter, the quality and composition of particulate matter have been shown to affect gut microbial assemblages. For example, Stevick et al. [75] reported that the microbiomes of oysters (*C. virginica*) displayed notable functional plasticity in response to eutrophication gradients, with shifts in the nutrient availability driving changes in the microbial structure and metabolic pathways. These findings emphasize the dynamic interplay between environmental nutrient inputs and host–microbiome interactions.

Though limited, emerging evidence in cephalopods suggests that carnivorous feeding strategies may promote distinct microbiome configurations. While most studies to date have been conducted in non-molluscan models (e.g., those by Masanja, Yang, Xu, He, Liu, Xu, Jiang, Luo, Mkuye, Deng and Zhao [3] and Michl et al. [76]), they provide conceptual frameworks indicating that protein-rich or variable diets enhance microbial diversity and functional resilience. However, given the phylogenetic and ecological differences, extrapolations to cephalopods must be interpreted cautiously. The lack of targeted studies in cephalopods highlights a critical knowledge gap, warranting future research on diet-driven microbial dynamics in these taxa.

The developmental stage of mollusks is another key factor modulating microbiome assembly. Transitions from the larval to juvenile and adult stages are typically associated with significant shifts in diet, habitat exposure, and immune maturation, each of which influences the microbial succession. Shoji et al. [77] demonstrated that the microbiome of juvenile bivalves differed markedly from that of adults, suggesting a phase-dependent restructuring of microbial communities. Such transitions may influence the nutrient assimilation efficiency, disease resistance, and developmental plasticity, reinforcing the microbiome’s integrative role in host life history strategies.

Recent studies have also explored dietary modulation as a means of enhancing microbiome stability. In particular, probiotic and prebiotic supplementation have been associated with improvements in the microbial composition, immune stimulation, and stress resilience in aquaculture-reared mollusks [78]. These findings suggest practical applications in aquaculture for promoting host health and productivity under fluctuating environmental conditions. Overall, the host’s diet and developmental stage exert synergistic effects on microbiome assembly and functionality. As such, they represent key levers for managing molluscan health in both natural and managed systems, while also offering testable hypotheses for future experimental validation, particularly in understudied groups such as cephalopods.

### 3.3. Habitat and Microhabitat Differences

Habitat and microhabitat variability exert substantial influence on the structure, composition, and functional dynamics of molluscan microbiomes. These spatial differences mediate microbial colonization by altering key environmental parameters, including the temperature, salinity, nutrient fluxes, substrate type, and biotic interactions. As a result, host-associated microbial communities often reflect the ecological conditions in which mollusks reside.

In aquatic systems, environmental heterogeneity has been shown to shape bivalve microbiomes, as seen in species such as *C. virginica*, where salinity gradients, thermal regimes, and the nutrient availability contribute to site-specific microbiota profiles [79]. Geographic variation in the microbiome structure across conspecific populations underscores the importance of local ecological drivers in shaping spatially differentiated microbial assemblages. This trend mirrors broader patterns of environmentally mediated microbiome divergence, which may underpin hosts’ adaptation to regional stressors. Research on built marine environments, including artificial substrates and aquaculture systems, further underscores the impact of anthropogenic influences on the microbiome composition. Zupičić et al. [80] demonstrated that modified habitats support distinct microbial taxa compared to natural substrates, with implications for microbial functionality and host health. These findings highlight the significance of the environmental context in shaping host–microbiome interactions, particularly in light of habitat transformation and urban coastal development.

Transitions between aquatic and terrestrial habitats, as seen in some gastropods, introduce additional complexity to microbiome dynamics. Changes in the humidity, light exposure, substrate chemistry, and desiccation risk can alter the colonization potential and survival of microbial taxa [81]. These abiotic shifts influence not only microbial diversity but also the functional contributions of symbionts to the host’s physiology. At finer spatial scales, microhabitat variation, such as localized differences within coral reefs or estuarine sediments, can also drive microbial differentiation. Li et al. [82] found that even within ecologically similar zones, subtle environmental gradients produce discrete microbiome profiles, suggesting that selective pressures at the micro-scale can shape microbial assembly. Although their study focused on coral-associated systems, similar microhabitat filtering likely applies to sessile mollusks, such as bivalves, whose microbiota are closely linked to their filter-feeding regimes and sediment contact.

Furthermore, a study on microhabitat interactions in migratory birds by Prestes et al. [83] suggests a possible parallel in mollusks. Filter-feeding mollusks are likely influenced by the site-specific food particle availability, which may generate microbiome variation across habitats; however, this remains underexplored and warrants direct investigation. Ultimately, the concept of phylosymbiosis provides an evolutionary framework for understanding microbiome divergence across different habitat types. This pattern, wherein the host’s phylogeny correlates with the microbial community structure, may interact with environmental factors to produce complex, habitat-mediated microbiome signatures even among closely related molluscan species [84]. Such findings suggest that ecological filtering and evolutionary history jointly shape molluscan microbiomes, a key consideration as climate change and habitat degradation continue to reconfigure marine and freshwater ecosystems. The environmental conditions and host lifestyle jointly influence the composition and functional capacity of molluscan microbiomes. Table 3 synthesizes the reported dominant microbial taxa, the environmental or host conditions under which they occur, and their inferred functional roles. This synthesis provides insight into adaptive microbiome responses to diverse ecological pressures.

### 3.4. Genetic and Species-Level Filters

The assembly of molluscan microbiomes is profoundly shaped by genetic and species-specific filters that serve as key biological determinants of the microbial community structure and function. These filters encompass diverse gene families regulating the complex interactions between the host and associated microbes. The host’s genetic makeup influences the microbiome composition through mechanisms including immune responses, physiological adaptations, and developmental gene expression. The evolutionary lineage also plays a pivotal role, as closely related species frequently harbor similar microbiomes, a pattern known as phylosymbiosis [87,88].

In mollusks, genetic filtering may result from variation in immune system function, metabolic pathways, and tissue-specific traits that selectively recruit microbial taxa. For example, differential immune gene expression significantly affects the microbial populations, with hosts exhibiting robust immune defenses maintaining beneficial microbiota while excluding pathogens [89,90]. The host’s genotype thus emerges as a major determinant of the microbiome composition across taxa. Supporting this, studies on marine phytoplankton demonstrate that the host’s evolutionary lineage strongly shapes bacterial communities and exerts selective pressure during microbiome assembly [89,91]. Beyond immune genes, research highlights specific gene families underpinning host–microbe interactions.

Mantle-specific genes are intimately linked to shell formation and resilience; Lopez-Anido et al. [92] identified lineage-restricted shell matrix proteins in *Crepidula atrasolea* Collin, 2003, implicating these proteins in shaping the shell morphology and influencing microbial colonization. Similarly, Bai et al. [93] showed that biomineralization-related genes contribute to the development of microbial assemblages associated with oyster shells, indicating a genetic–environmental interface shaping the microbiome composition. Developmental genes, such as those in the Hox family, also contribute to genetic filtering by dictating the body plan and tissue architecture. Variations in Hox gene expression correlate with structural differences among molluscan species, indirectly modulating microbial communities by altering the habitat niches within the host [94,95]. These genes guide morphological diversification and influence the physical environment available for microbial colonization. Immune-related gene families remain critical modulators of host–microbiome dynamics. Schultz and Adema [96] demonstrated that variation in immune gene expression produces diverse host defense strategies, impacting the microbial community structure. Liu et al. [97] further linked immune genetic variation to microbiome diversity, emphasizing the role of immunity-associated loci in shaping gut microbial assemblages. Metabolic genes form another axis influencing microbiomes. Liu et al. [98] investigated the GATA2/3 gene in *C. gigas*, suggesting its involvement in shell formation and related metabolic processes that potentially affect microbiome structuring. Additionally, the orthologous shell-forming proteins characterized by Jackson et al. [99] provide insight into how genetic variation governs phenotypic traits linked to microbial diversity.

**Figure 2:**
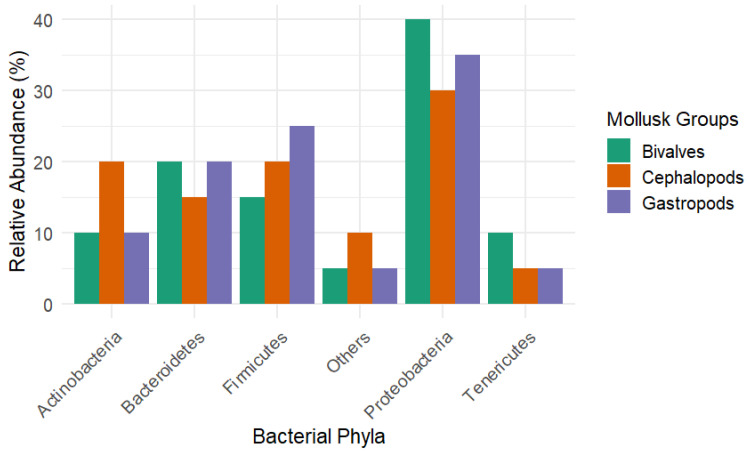
Comparative bar chart showing dominant bacterial phyla across gastropods, bivalves, and cephalopods.

Genomic studies reveal unique expansions in molluscan gene families corresponding to environmental adaptations, reinforcing the central role of the host’s genetics in microbiome assembly [100]. Collectively, these findings underscore that molluscan genetic backgrounds—including immune, developmental, metabolic, and biomineralization genes—orchestrate selective microbial recruitment and shape distinct microbial landscapes. Moreover, species-specific traits shaped by evolutionary divergence influence microbial colonization patterns. Closely related species tend to support similar microbiomes due to shared ecological niches and genetic heritage [88]. However, the interplay between intrinsic genetic factors and extrinsic environmental conditions collaboratively drives microbial diversity and assembly mechanisms [101,102]. Finally, heritable traits linked to microbial community structures extend beyond mollusks to other taxa. For example, studies in cattle demonstrate associations between heritable microbiome traits and metabolic efficiency, suggesting co-evolutionary dynamics between the host’s genetics and microbiota [103,104]. Similar patterns may occur in mollusks, where genetically influenced traits such as the shell morphology and feeding strategies facilitate species-specific microbial signatures [88,105]. Differences in the microbiome composition are evident across mollusk classes, reflecting their evolutionary history and ecological adaptations. Figure 2 compares the relative abundance of dominant bacterial phyla among gastropods, bivalves, and cephalopods, highlighting class-specific microbial signatures. Such taxonomic distinctions support the concept of phylosymbiosis and the role of the host’s phylogeny in microbiome assembly.

## 4. Functional Contributions of the Molluscan Microbiome

### 4.1. Taxon-Specific Functional Roles in Molluscan Metabolism

Recent studies have increasingly emphasized the relationship between molluscan taxa and their metabolic processes, revealing critical insights into ecological adaptations and physiological diversity. According to the Metabolic Theory of Ecology (MTE), the metabolic rate serves as a central driver of ecological processes, shaping organismal function across various scales [106,107]. This theory is particularly relevant to mollusks, where the metabolic rates have been shown to vary widely across taxa, even within closely related groups such as bivalves [108,109]. For instance, species-specific differences in the hypoxia tolerance among bivalves are closely linked to variations in metabolic pathways and the accumulation of specific metabolites, enabling survival in oxygen-poor environments [108]. Similarly, habitat-specific adaptations among mollusks reflect divergent metabolic strategies that have evolved to accommodate environmental stressors such as fluctuations in the temperature and oxygen availability [110].

At the biochemical level, mollusks utilize a range of metabolic processes for energy production. These include both aerobic and anaerobic respiration, with lipid metabolism playing a key role in energy storage, particularly during reproduction or thermal stress [111,112]. In marine mollusks, amino acid metabolism not only provides energy but also supports osmoregulation, highlighting the functional duality in metabolic roles. Anaerobic pathways are especially important in taxa inhabiting hypoxic or benthic environments, enabling sustained activity under limited oxygen conditions. For example, terrestrial snails rely heavily on carbohydrate reserves such as galactogen, which are critical during embryonic development, whereas gastropods in marine and freshwater systems display a broader spectrum of metabolic flexibility [112].

Taxonomic differences in metabolism have implications beyond physiology, influencing how mollusks contribute to ecosystem processes like nutrient cycling and habitat formation [110,113,114]. From an ecological perspective, metabolic rates shape trophic interactions and community dynamics, making them central to understanding molluscan roles in aquatic and terrestrial ecosystems. Furthermore, in the context of climate change, the metabolic sensitivity to stressors such as ocean acidification and warming is increasingly used to predict molluscan resilience and vulnerability [115,116,117]. Understanding the interplay between taxonomy and metabolism is thus essential not only for elucidating molluscan evolutionary history but also for informing conservation strategies in rapidly changing environments.

### 4.2. Digestion, Metabolism, and Nutrient Cycling

The molluscan microbiome plays a critical and multifaceted role in the host’s physiology, particularly in digestion, energy metabolism, and broader nutrient cycling within aquatic ecosystems. Symbiotic microbial communities enable mollusks to break down complex dietary components, synthesize essential nutrients, and participate in biogeochemical processes that benefit both the individual host’s health and ecosystem functioning. Specific microbial taxa, including genera such as *Photobacterium* and *Clostridium*, dominate the molluscan gut and are known to produce specialized enzymes like cellulases and hemicellulases that degrade structural carbohydrates such as cellulose and hemicellulose [118,119,120]. For example, *Clostridium thermocellum* produces cellulosomes, multiprotein complexes that efficiently convert cellulose into fermentable sugars. These sugars are then fermented by gut microbiota into short-chain fatty acids (SCFAs) such as acetate, propionate, and butyrate, which serve as pivotal energy substrates for the host [121]. Quantitative studies reveal that SCFAs can meet up to 30% of the host’s energy needs and regulate metabolic and immune pathways via G-protein-coupled receptors [122,123]. Experimental disruption of molluscan microbiota, for instance through antibiotic treatment in bivalves like *M. edulis*, has been shown to impair cellulose degradation and reduce the host’s energy intake, underscoring the indispensable role of microbial communities in nutrient assimilation and host growth [123,124]. Moreover, microbial contributions to biosynthesis of vitamins and amino acids enhance the host’s nutritional status, improving its resilience against environmental stress and disease [125]. Beyond individuals’ physiology, molluscan microbiomes profoundly influence ecosystem nutrient cycling. Filter-feeding bivalves capture suspended organic matter, with associated microbes mineralizing these compounds to release nitrogen and phosphorus in bioavailable forms for primary producers. This process supports aquatic primary productivity and water quality regulation, positioning mollusks as key ecological engineers in coastal and estuarine environments [126,127]. Microbiome-mediated nutrient transformations link molluscan feeding strategies to broader carbon and nitrogen cycles critical for ecosystem stability. Understanding these functional host–microbiome relationships has practical implications for aquaculture and environmental management. Manipulating molluscan microbiomes via mollusks’ diet or probiotics to enhance beneficial microbial taxa could improve the growth rates, disease resistance, and feed efficiency, thereby optimizing production and sustainability in mollusk farming [128]. Simultaneously, recognizing microbial roles in nutrient cycling aids conservation efforts, especially as climate change and anthropogenic pressures disrupt aquatic food webs and microbial community structures [129].

### 4.3. Immune Modulation and Pathogen Defense

Molluscan microbiomes play a key role in modulating host immune responses and defending against pathogens. This enhances the host’s resilience under environmental stress. These effects arise from immunostimulation, biochemical antagonism, and ecological interactions within microbial communities. Mollusks rely solely on innate immunity, including hemocytes (immune cells), antimicrobial peptides (AMPs), and signaling molecules. Recent studies have shown that gut and mucosal microbiomes actively regulate these immune components, rather than just passively existing [130,131]. For example, diverse mucosal microbes in bivalves promote hemocyte proliferation and AMP expression, strengthening immunity. Experiments in oysters confirmed that healthy microbiota improve the infection resistance, suggesting microbially mediated immune training (Allam and Espinosa [130]). The interaction between the gut microbiota and immune pathways—especially those relating to Toll-like receptor (TLR), immune deficiency (IMD), and the Janus kinase/signal transducer and activator of transcription (JAK/STAT)—is crucial for host defense. These pathways control AMP production, hemocyte activation, and melanization. The TLR and IMD pathways detect microbial signals and trigger immune responses. The IMD pathway is well-studied in invertebrates like Drosophila, where gut bacteria stimulate AMP production for frontline defense [74,132]. Disrupting the gut microbiota impairs IMD pathway activation, reducing the AMP output and highlighting their symbiotic link [133,134]. The JAK/STAT pathway regulates inflammation and tissue repair. Its activation boosts the AMP levels and hemocyte activity, key in achieving innate immunity [74,135]. It also contributes to melanization, a process which encapsulates and neutralizes pathogens [132,136]. The gut microbiota can modulate the JAK/STAT pathway, creating a feedback loop that shapes immune responses and the microbial balance [136,137]. Some commensal bacteria, such as *Pseudomonas* spp., produce antimicrobial compounds like phenazines and siderophores, directly protecting the host and stabilizing the microbiome [138]. Other microbes produce bacteriocins, lytic enzymes, or short-chain fatty acids that inhibit pathogens chemically [139,140]. These products reduce the pathogen loads in tissues like the gills, gut, and hemolymph, forming barriers against disease. Microbiomes also defend their hosts through competitive exclusion, where beneficial microbes outcompete pathogens for space and nutrients. Studies in bivalves show that resident microbes form stable biofilms on their gills and digestive tracts, preventing opportunistic infections (Ellison et al. [141,142]). This layered defense and functional redundancy promote system stability despite environmental changes. However, environmental stressors such as warming, acidification, and pollution can disrupt microbial communities. This leads to dysbiosis, reduced immune stimulation, and greater pathogen susceptibility. For example, Ye et al. [143] showed that dietary and environmental stressors in pearl oysters (*Pinctada fucata martensii* and *P. maxima*) impaired feeding and metabolic efficiency, reduced beneficial microbial functions, and favored pathogenic taxa, thereby compromising host resilience. Similarly, Berg and Koskella [144] demonstrated that host-associated microbial communities confer pathogen protection in a nutrient- and dose-dependent manner, but this protection collapses under altered environmental conditions. Environmental degradation increases infections by pathogens like *Perkinsus* spp., causing mass mortalities. Maintaining diverse, healthy microbiota may buffer these effects by supporting the immune balance and lowering the pathogen burden. This highlights the role of microbiome health in conservation, aquaculture, and disease control. Environmental factors such as antibiotics and diet changes disrupt gut microbiota, causing dysbiosis that weakens immune pathways. Antibiotic treatments reduce microbiota diversity, lowering IMD and JAK/STAT pathway activation, and thus impair AMP production and hemocyte function [133,134]. This immune dysregulation increases the infection risk and delays healing, emphasizing the need for balanced microbiomes to maintain optimal immunity [137].

### 4.4. Sulfur/Nitrogen Cycling and Toxin Handling

In nitrogen cycling, molluscan microbiomes contribute to processes involved in multiple transformation pathways, including nitrogen fixation, nitrification, and the oxidation of ammonia. Black, Chimenti and Just [5] and Gillikin et al. [145] revealed the presence of functionally relevant bacterial taxa within bivalve gut and gill microbiomes capable of converting nitrogenous compounds into bioavailable forms. These processes enhance the nutrient availability in oligotrophic waters, contributing to the nitrogen budget of coastal systems. The ability of these microbiota to transform nitrogenous waste also reduces the accumulation of toxic ammonia within host tissues, providing a physiological benefit to the mollusks themselves. Such microbial activities establish bivalves not only as participants in benthic–pelagic coupling but also as valuable bioindicators of the nitrogen dynamics in aquatic environments.

Sulfur metabolism represents another critical ecological function facilitated by the microbiomes of mollusks. Sulfate-reducing bacteria (SRB), often found in association with bivalve tissues or in the surrounding sediment influenced by mollusk activity, are integral to the sulfur cycle. These microbes contribute to the transformation of sulfate into hydrogen sulfide, regulating the sediment chemistry and maintaining the redox balance [145]. The symbiotic presence of SRB may also mitigate the accumulation of potentially harmful sulfur compounds within mollusk tissues, reflecting a detoxification benefit aligned with environmental regulation.

Beyond nutrient cycling, mollusks regularly encounter a wide range of environmental toxins, including heavy metals, pesticides, and biotoxins produced during harmful algal blooms (HABs). Microbial symbionts contribute to host detoxification by sequestering these toxicants or transforming or degrading them into less bioavailable forms. Rossignoli et al. [146] and Zan et al. [147] have demonstrated that specific gut microbes play a role in metabolizing xenobiotic compounds, enhancing molluscan resilience under pollutant exposure. In the context of HABs, species such as *C. gigas* and *M. galloprovincialis* exhibit microbiome-mediated detoxification of neurotoxins, including domoic acid and okadaic acid. Studies by Blanco et al. [148] and Valdiglesias et al. [149] confirmed that microbial activity aids in degrading or transforming these compounds, thereby reducing their physiological burden on the host.

Microbiome-driven biotransformation of paralytic shellfish toxins (PSTs) has also been observed. Specific bacterial taxa are capable of converting PSTs into less toxic analogs, potentially altering the risk profile for both mollusks and their predators [150,151]. This microbial function not only promotes host survival but also has cascading implications for food safety and the stability of the marine food web. The combined contributions of molluscan microbiomes to nitrogen/sulfur cycling, as well as toxin mitigation, underscore their dual ecological and physiological significance. From an applied perspective, these functions offer actionable insights for aquaculture and environmental monitoring. For instance, manipulating microbiomes using dietary interventions or probiotics may enhance hosts’ resistance to environmental toxins and improve the nutrient cycling efficiency in farming systems [152,153]. Further research is warranted to identify key microbial taxa responsible for these functions and to explore their biotechnological potential for use in ecosystem restoration and pollution mitigation.

### 4.5. Functional Redundancy and Stability

Functional redundancy within molluscan microbiomes is increasingly recognized as a vital mechanism supporting host resilience and ecosystem stability. This concept reflects the ability of diverse microbial communities associated with mollusks to maintain essential ecological and physiological functions even when their taxonomic composition shifts. Foundational work in marine invertebrates, including corals, has shown that microbiomes can uphold critical processes like nutrient cycling and pathogen suppression despite changes in the microbial community structure [154]. Similar principles have been confirmed in mollusks, where phylogenetically distinct bacteria perform overlapping functions that help stabilize the host biology under environmental stress [56].

The microbial taxa in both bivalves and gastropods contribute to key physiological processes such as digestion, detoxification, immune modulation, and nutrient assimilation. Studies indicate that many of these taxa possess metabolic redundancy, meaning that different species share similar functional genes or biochemical pathways [155]. For example, Szabó et al. demonstrated that microbial communities in gastropods can shift without the loss of essential physiological functions, highlighting redundancy as a buffer against dysbiosis. This capacity enables mollusks to maintain homeostasis despite fluctuating environmental conditions [156,157]. Complementing these findings, metaproteomic and metagenomic analyses reveal that diverse bacterial lineages share metabolic outputs such as fermentation, oxidative stress mitigation, and immune regulation, further contributing to the system’s resilience [56,158].

Though direct causal links between specific redundant taxa and toxin detoxification remain limited, evidence suggests that multiple bacterial groups collaboratively transform harmful compounds, especially in bivalves exposed to toxins from harmful algal blooms [56]. These insights point to the value of multi-omics approaches for elucidating the functional gene networks that sustain redundancy and host protection. Beyond the host’s physiology, functional redundancy influences broader ecosystem processes, including nitrogen cycling, sulfur metabolism, and organic matter decomposition. These microbial activities regulate aquatic biogeochemistry and enhance trophic interactions, maintaining ecosystem health even under environmental stress [159,160]. The persistence of such functions underlines the stabilizing role of molluscan microbiomes in aquatic environments.

**Figure 3:**
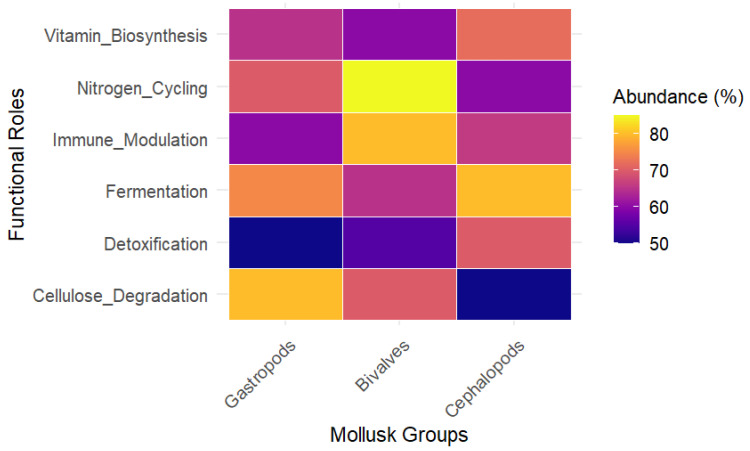
Heatmap of microbial functional gene categories associated with different molluscan hosts, including genes involved in digestion, nutrient cycling, immune modulation, and stress resilience.

From an evolutionary perspective, functional redundancy likely reflects co-adaptive relationships between mollusks and their symbiotic microbes. This mutualism allows hosts to preserve essential physiological functions over time despite a turnover in microbial partners, conferring adaptive advantages in variable habitats and supporting long-term fitness and population stability [161,162]. Specific taxon examples underscore these concepts. The gastropod *A. ater* relies on gut microbes for lignocellulose digestion, with functional redundancy among bacterial taxa ensuring continued nutrition despite microbial shifts [4]. In the bivalve *M. edulis*, multiple bacterial taxa help mitigate oxidative stress by controlling the production of reactive oxygen species under environmental challenges, supporting host health in dynamic marine settings [163]. Important microbial genera such as *Nitrospira* and *Nitrosomonas* drive nitrogen cycling, while *Desulfovibrio* species contribute to sulfur metabolism, illustrating ecological functional redundancy. Additionally, core bacteria like *Cloacibacterium* and *Aeromonas* show persistent transmission and multifunctionality across gastropods [1]. To better characterize the functional landscape of molluscan microbiomes, Figure 3 presents a heatmap depicting microbial gene categories associated with digestion, nutrient cycling, immune responses, and stress resilience. This visualization highlights both functional redundancy and specialization across microbial communities, underpinning hosts’ physiological processes and ecosystem functions.

## 5. Applications in Aquaculture, Conservation, and Environmental Monitoring

### 5.1. Health Monitoring and Bioindicators

The application of microbiome research in aquaculture has emerged as a transformative strategy for enhancing the sustainability, health, and productivity of farmed mollusks and fish. Microbial communities associated with aquaculture species function as dynamic indicators of the host’s physiology and environmental integrity, offering diagnostic potential for early detection of disease, stress, and ecosystem imbalances. This functional relevance positions microbiome profiling as an essential tool in adaptive aquaculture management [27,164]. Microbial communities are integral to hosts’ health, reflecting both their internal physiological status and external environmental conditions. Perturbations in the microbial composition frequently precede visible symptoms of disease or ecological stress. For instance, Hines, Madanick, Smith, Kuhn and Stevens [27] demonstrated that alterations in the gut microbiota of *C. virginica* were correlated with pathogen exposure and pollutant stress, reinforcing the utility of microbiome profiles as early-warning biomarkers. Similarly, Green et al. [165] found that shifts in the microbial diversity in farmed oysters mirrored broader environmental disruptions, suggesting that microbiomes can serve as sensitive bioindicators of the health of aquaculture systems.

Beyond passive monitoring, molluscan microbiomes actively contribute to disease resistance. Beneficial taxa play crucial roles in pathogen exclusion through competitive niche occupation, the production of antimicrobial peptides, and the stimulation of host immunity. For example, Clols-Fuentes et al. [166] observed that fish-associated skin microbiota suppressed opportunistic pathogens by maintaining microbial homeostasis and producing inhibitory metabolites. Although this study focused on finfish, similar microbial dynamics have been identified in mollusks, where microbiota in the gills, gut, and mantle tissues contribute to immune modulation and pathogen defense [130,142]. Environmental drivers, such as the temperature, nutrient enrichment, and pollutants, exert a significant influence on host-associated microbial communities. Green, Siboni, King, Labbate, Seymour and Raftos [165] reported that increased sea surface temperatures led to an elevated *Vibrio* Pacini, 1854, abundance, preceding disease outbreaks in Pacific oysters. These findings underscore the predictive value of microbiome shifts in managing aquaculture health risks and suggest that environmental microbiome surveillance could anticipate pathogen emergence and ecological imbalances.

Technological advances in high-throughput sequencing and bioinformatics now allow for routine microbiome monitoring within aquaculture operations. By establishing baseline microbial profiles and tracking the deviations over time, aquaculturists can detect dysbiosis or pathogen colonization before clinical symptoms arise. Wen, Zuccarello, Klochkova and Kim [164] advocate for integrating such microbial diagnostics into standard aquaculture management practices, enabling interventions such as feed adjustments, water quality optimization, or probiotic supplementation. These strategies not only promote host resilience and health but also reduce the need for antibiotics, supporting a more sustainable and ecologically responsible aquaculture model [152,153].

### 5.2. ARGs, Food Safety, and Microbiome Manipulation

The intensification of antibiotic use in aquaculture has caused a significant rise in antimicrobial resistance (AMR), threatening aquatic animals’ health, food safety, and public health. Aquaculture systems now serve as reservoirs of antibiotic resistance genes (ARGs). Su et al. [167] reported 492 ARG subtypes across various farming environments. The spread of these ARGs reduces treatments’ effectiveness and increases the risk of horizontal gene transfer to human pathogens. This situation highlights the urgent need for surveillance and mitigation. Microbiome profiling is a promising method to monitor ARG emergence in aquaculture. Changes in microbial communities correlate with the ARG prevalence, suggesting that gut microbiota analysis can serve as an early warning system [168]. Routine genomic surveillance with metagenomic tools helps detect ARGs and assess microbial responses to antibiotics. This information supports adaptive management strategies to reduce antibiotic use and enhance system resilience. Food safety depends heavily on the microbial dynamics in aquaculture. Pathogenic bacteria carrying ARGs can grow in poor farming conditions, contaminating edible tissues and risking public health. Targeted microbiome monitoring in key species such as *Oncorhynchus mykiss* (Walbaum, 1792) (rainbow trout) and *Paralichthys olivaceus* (Temminck & Schlegel, 1846) (olive flounder) is essential to identify harmful taxa and enforce biosecurity measures [169,170]. Advances in next-generation sequencing allow for rapid detection of microbial hazards, improving hygiene and handling practices across the supply chain. Manipulating the microbiome using probiotics and specific diets is a sustainable way to combat AMR and boost aquaculture productivity. Probiotics like *Lactobacillus plantarum* Orla-Jensen, 1919, modulate gut microbiota, enhance immune responses, and reduce antibiotic reliance in farmed fish [169]. These microbes also competitively exclude pathogens, improve nutrient use, and stabilize the system. Nutritional strategies replacing traditional proteins with novel feed ingredients promote beneficial microbiome shifts and better growth performance [171]. These findings emphasize the need to integrate microbiome management into feed formulation and aquaculture practices to improve animal health and reduce the ecological and public health risks from AMR.

Recent studies have revealed specific classes of ARGs prevalent in molluscan microbiomes and associated environments. These include β-lactamase genes such as *bla_CTX-M*, tetracycline resistance genes (*tetA*, *tetM*), the macrolide resistance gene *ermB*, sulfonamide resistance genes (*sul1*, *sul2*), and aminoglycoside resistance genes like *aadA* [172,173,174]. Sulfonamide resistance genes, particularly *sul1* and *sul2*, often dominate in aquaculture sediments, reflecting strong anthropogenic impacts [175]. Tetracycline resistance genes (*tetA*, *tetM*) are also commonly detected in farm-associated microbial communities [172,174]. The presence of these ARGs compromises antibiotic efficacy, resulting in treatment failures for infections in aquaculture and potentially in human medicine. Moreover, horizontal gene transfer from mollusks or their microbiomes to human pathogens poses significant public health concerns [163]. The use of detection methods such as quantitative polymerase chain reaction (qPCR) and metagenomic analyses has clarified the prevalence and diversity of ARGs in aquaculture sediments and molluscan microbiomes [176,177]. For example, Naquin and Boopathy [172] successfully used a qPCR to detect *sul1* and *sul2* genes, confirming their widespread presence in aquaculture environments. Understanding the specific ARG types and their distributions allows for targeted surveillance and microbiome-based interventions. Identifying ARGs associated with pathogens commonly found in mollusks, such as *Vibrio* spp. and *Escherichia coli*, supports the development of improved biosecurity measures and management practices aimed at reducing the AMR risks [178]. Integrating these insights into aquaculture management is essential to safeguard food safety and public health while promoting sustainable production.

### 5.3. Probiotics, Biomarkers, and Sustainable Aquaculture

The integration of probiotics, health biomarkers, and advanced environmental monitoring technologies is reshaping aquaculture into a more sustainable, precision-driven, and health-conscious industry. These interventions promote improved animal welfare, reduced antibiotic dependence, and enhanced ecosystem stewardship, core priorities in modern aquaculture practice.

Probiotics, defined as live microorganisms that confer health benefits on the host, are increasingly applied in aquaculture to stabilize the gut microbiome, stimulate immune responses, and reduce the pathogen load. Their use has been associated with improved growth rates, enhanced feed conversion efficiency, and lowered mortality in various molluscan and finfish species [171,179]. For instance, *L. plantarum* has demonstrated immunomodulatory and digestive benefits in shrimp and teleost fish, contributing to disease resistance and nutrient absorption. Multi-strain probiotic formulations are particularly promising due to their broader functional scope, enhancing microbial diversity, enzyme activity, and systemic immunity [180]. By offering a viable alternative to antibiotics, probiotics also support antimicrobial stewardship and the production of safer seafood [179]. Probiotics also contribute to environmental sustainability through microbial degradation of organic waste and suppression of harmful microbial blooms. These dual benefits improve the water quality and reduce the need for intensive chemical management, aligning aquaculture with ecological conservation goals [181,182]. Environmental monitoring technologies, particularly remote sensing and satellite-based assessments, facilitate the tracking of nutrient loads, pollution levels, and land use change at multiple scales [183,184]. Probiotic application in mollusk aquaculture is technically feasible using several practical methods that have shown promise in other aquatic systems. One approach involves the direct addition of probiotic cultures, such as *L. plantarum*, into the rearing water, allowing for interaction with the mollusks’ external surfaces and potential colonization of epithelial tissues [185,186,187]. Another widely used method is coating feed pellets with probiotic formulations using biocompatible binders (e.g., alginate or starch-based polysaccharides), enabling direct delivery to the digestive system upon ingestion [186]. A third technique includes incorporating probiotics into formulated supplemental feeds, which ensures consistency in the dosage and can be optimized to deliver synergistic nutritional benefits [188]. Critical to the success of these interventions is the selection of strains with a proven ability to colonize molluscan guts, produce antimicrobial compounds, and stimulate immune responses. Moreover, monitoring the outcomes through microbial profiling (e.g., 16S rRNA gene sequencing) and health indicators like the growth rates, survival, and disease incidence enables iterative refinement of probiotic treatments [173]. Collectively, these strategies offer a promising framework for applying probiotics in mollusk farming, though species-specific validation remains essential.

Biomarkers have emerged as vital tools for monitoring the physiological condition of cultured organisms and predicting disease susceptibility. Conventional biomarkers, such as cortisol, lysozyme activity, and heat shock proteins, enable the real-time detection of stress, immune activation, and environmental perturbations [189,190]. Technological advancements have introduced non-invasive biosensors capable of continuous monitoring of both the host’s physiology and environmental variables. These biosensors detect critical indicators such as toxins, the dissolved oxygen levels, and pathogenic signatures in aquaculture water, enabling early intervention and reducing the reliance on chemical treatments [191,192]. Furthermore, real-time detection of harmful algal blooms and salinity fluctuations via biosensing systems enhances operational safety and biosecurity [174]. The integration of Internet of Things (IoT) systems, often referred to as “smart aquaculture,” enables the automated regulation of key environmental parameters, including the temperature, pH, and dissolved oxygen. These systems support precision farming by enabling real-time decision-making, efficient resource utilization, and early detection of suboptimal conditions [193,194]. Collectively, these innovations highlight the pivotal role of microbiome-informed practices and digital tools in fostering sustainable, resilient, and environmentally responsible aquaculture systems.

## 6. Future Directions

A central limitation is the lack of standardized methodologies for sample collection, DNA extraction, sequencing, and bioinformatics pipelines. While 16S rRNA gene sequencing enables broad community profiling, inconsistencies in study designs hinder the comparability and reproducibility across taxa and systems [27,195]. Furthermore, amplicon-based studies exclude non-bacterial taxa and lack functional resolution. Adoption of multi-omics approaches—such as metagenomics, metatranscriptomics, metabolomics, and metaproteomics—is crucial for holistic characterization of the microbiome function and dynamics [196,197], as fungal and archaeal taxa may participate in immune regulation and nutrient cycling yet remain poorly characterized. Expanding analyses beyond bacterial taxa will offer a more ecologically realistic view of the microbial networks in mollusks. Even among bacteria, several key genera—such as *Alcanivorax*, *Vibrio*, and *Pseudomonas*—are recurrently detected but undercharacterized functionally in mollusks [198]. Predictive tools like PICRUSt2, HUMAnN3, and KEGG orthology pipelines should be systematically employed to infer potential microbial functions, followed by culture-based validation [171]. The host’s genetics, evolutionary history, and habitat specificity are additional factors influencing the microbiome composition, yet few studies integrate host transcriptomes or immune gene expression profiles with microbial data [170]. A combined eco-evolutionary framework is needed to understand how host filters shape microbial colonization, persistence, and co-evolution. Finally, while microbial monitoring and probiotic-based interventions show promise for use in aquaculture, their application is limited by their cost, a lack of field validation, and their scalability. Most studies remain laboratory-bound, with short-term endpoints. Longitudinal, in situ trials are needed to assess the effects of microbiome manipulation on host health, microbial stability, ecosystem resilience, and antimicrobial resistance [199]. The development of portable diagnostic tools and training programs for aquaculture practitioners is equally essential for real-world implementation [27]. Advancing molluscan microbiome research through interdisciplinary, integrative approaches will not only enhance our understanding of host–microbe co-evolution but also unlock novel applications for sustainable aquaculture, conservation translocations, and environmental biomonitoring.

## 7. Conclusions

Molluscan microbiomes play multifaceted roles in host physiology, health, and adaptation across diverse aquatic and terrestrial ecosystems. This review consolidates the current evidence on microbial diversity and function in gastropods, bivalves, and cephalopods, revealing both conserved and lineage-specific patterns shaped by the ecological context and host biology. Despite notable progress, critical knowledge gaps constrain the full integration of microbiome insights into aquaculture, conservation, and environmental monitoring.

## Figures and Tables

**Table 1:** Summary of dominant bacterial phyla, functional roles, environmental sensitivities, and representative studies across major molluscan groups.

Host Species	Tissue Type	Dominant Taxa (Genus/Family)	Putative Functional Role	Environmental Sensitivity	Reference
*M. galloprovincialis*	Gut, gills	*Vibrio* (Vibrionaceae)	Chitin degradation, carbohydrate metabolism	Temperature-sensitive	[29]
*A. fulica*	Gut	*Lactobacillus* (Lactobacillaceae)	Lactic acid fermentation, pathogen suppression	pH-sensitive	[4]
*O*. *vulgaris*	Skin, gut	*Mycoplasma* (Mycoplasmataceae)	Immune modulation, amino acid biosynthesis	Sensitive to salinity and stress	[37]
*Crassostrea gigas* (Thunberg, 1793)	Gills, mantle	*Pseudoalteromonas* (Alteromonadaceae)	Antimicrobial production, biofilm regulation	Pollution-tolerant	[38]
*Ruditapes philippinarum* (Adams & Reeve, 1850)	Gut	*Pseudomonas* (Pseudomonadaceae)	Iron metabolism, detoxification	Oxygen-sensitive	[39]
*Haliotis discus hannai* (Ino, 1953)	Digestive gland	*Endozoicomonas* (Hahellaceae)	Host–symbiont communication, vitamin synthesis	Temperature-sensitive	[40]
*Lottia gigantea* (Gmelin, 1791)	Foot tissue	*Roseobacter* (Rhodobacteraceae)	Sulfur cycling, biofilm formation	Sensitive to metal stress	[41]

**Table 2:** Key features influencing molluscan microbiomes.

Feature	Description	Examples from Studies	References
Host Species Influence	Different mollusk species harbor unique microbial communities.	Studies comparing gut microbiota of different mussel species show significant differences in community structure.	[56,57,58]
Phylogeny	The host’s phylogeny is a major factor shaping the gut microbiome.	Cephalopods have distinct gut microbial communities compared to other mollusks, linking evolutionary history and microbiota.	[2,32]
Habitat	Different habitats lead to variations in microbial composition.	Differences between mangrove and island populations of same mollusk species highlight habitat’s role.	[59,60,61]
Diet	Their diet influences the microbial makeup of mollusks.	Freshwater gastropods share core gut microbes like *Aeromonas* and *Cloacibacterium*, suggesting adaptation to food sources.	[1,62]
Environmental Factors	Environmental conditions such as ocean acidification alter microbial diversity within species.	Ocean acidification decreases gut microbial diversity and immunity in oysters, showing environmental impacts on microbiomes.	[63,64]
Functional Significance	Distinct microbial signatures associated with phenotypes or disease resistance.	Differences in beta diversity between infected and uninfected snails suggest microbiome’s involvement in disease susceptibility.	[64,65]

**Table 3:** Summary of conditions, dominant microbial taxa, and functional roles in molluscan microbiomes.

Mollusk Group/Habitat	Dominant Taxa (Genus/Family)	Conditions/Influences	Reported or Inferred Functional Role(s)	References
*M. galloprovincialis* (marine)	*Vibrio*, *Mycoplasma*, *Pseudomonas*	Environmental salinity, pollutants	Immune modulation, digestion	[66]
*A. fulica* (terrestrial)	*Enterobacteriaceae*, *Bacillus*	Host’s diet, urbanization	Cellulose degradation, vitamin production	[54]
*S. officinalis* (gut)	*Photobacterium*, *Mycoplasma*	Starvation, dietary shifts	Nutritional plasticity, energy acquisition	[4,85]
*O. vulgaris* (skin)	*Pseudomonas*, *Alteromonas*	Sex-specific variation, ink secretion	Pathogen defense, skin barrier integrity	[37,39]
*C. gigas* (estuaries)	*Arcobacter*, *Vibrio*	Temperature stress, metal pollution	Community shifts under stress, disease susceptibility	[86]
*Octopus mimus* Gould, 1852 (reproductive tissues)	*Mycoplasma*	Environmental toxins, low diversity	Reproductive success, vertical transmission	[68,69]

## Data Availability

The data are provided within the body of the article.
